# Research progress of electrochemical oxidation and self-action of electric field for medical wastewater treatment

**DOI:** 10.3389/fmicb.2022.1083974

**Published:** 2023-01-06

**Authors:** Jun Tang, Heng Zheng, Jinzhong Cai, Jiang Liu, Yangyang Wang, Jun Deng

**Affiliations:** ^1^Department of Neurothoracic Surgery, The Third People's Hospital of Hubei Province Yangluo Campus, Jianghan University, Wuhan, China; ^2^College of Resources and Environmental Engineering, Wuhan University of Technology, Wuhan, China; ^3^Department of Interventional Radiology, Shenzhen People's Hospital (The First Affiliated Hospital, Southern University of Science and Technology), Shenzhen, China; ^4^College of Chemistry, Chemical Engineering and Life Sciences, Wuhan University of Technology, Wuhan, China; ^5^Department of Emergency, The Third People's Hospital of Hubei Province, Jianghan University, Wuhan, China

**Keywords:** medical wastewater, disinfection, electrochemical oxidation, electrolytic chlorination, self-action of electric field

## Abstract

A large number of pathogenic microorganisms exist in medical wastewater, which could invade the human body through the water and cause harm to human health. With the global pandemic coronavirus (COVID-19), public health safety become particularly important, and medical wastewater treatment is an important part of it. In particular, electrochemical disinfection technology has been widely studied in medical wastewater treatment due to its greenness, high efficiency, convenient operation, and other advantages. In this paper, the development status of electrochemical disinfection technology in the treatment of medical wastewater is reviewed, and an electrochemical three-stage disinfection system is proposed for the treatment of medical wastewater. Moreover, prospects for the electrochemical treatment of medical wastewater will be presented. It is hoped that this review could provide insight and guidance for the research and application of electrochemical disinfection technology to treat medical wastewater.

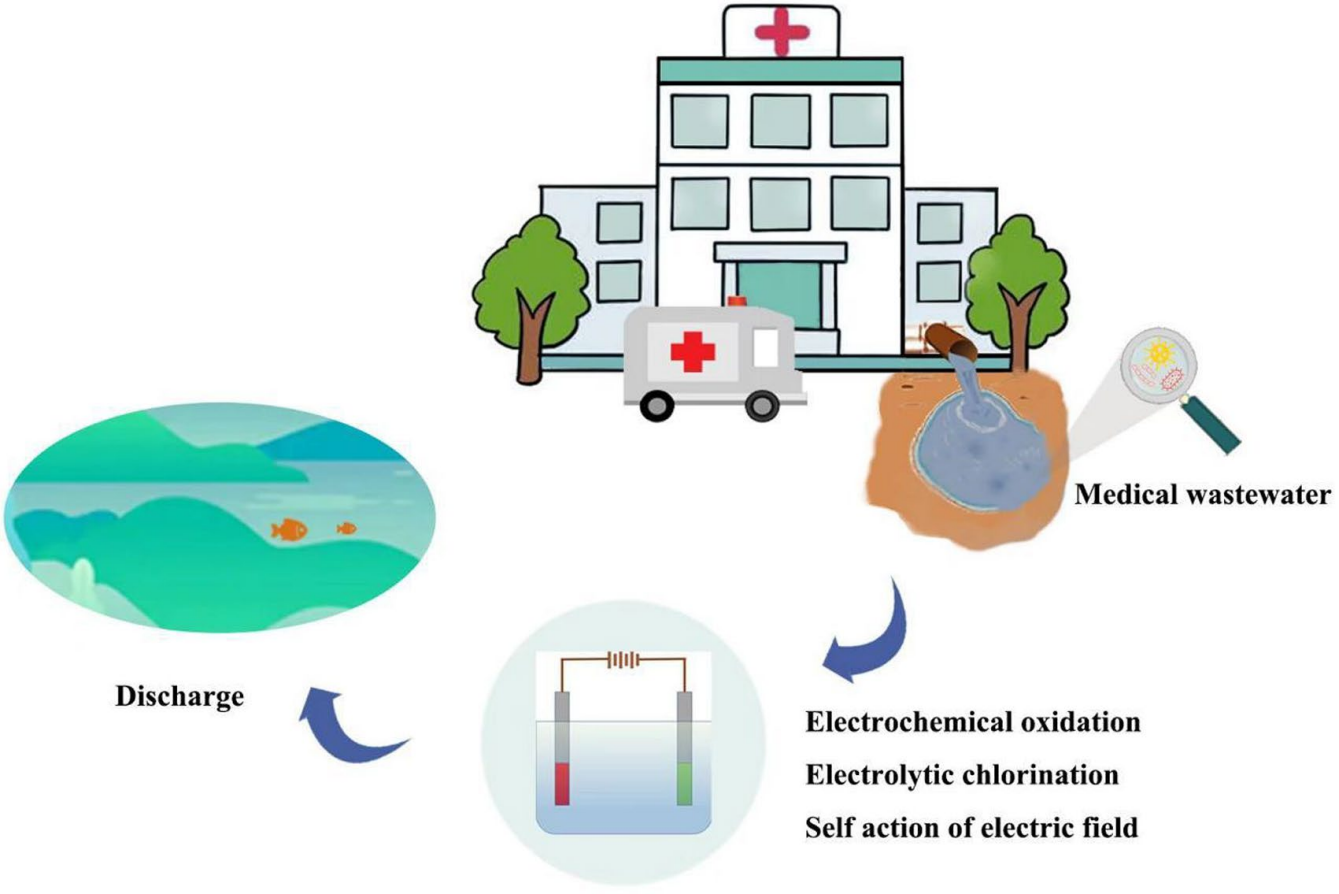

## 1. Introduction

Medical wastewater treatment is an important part of epidemic prevention and control and urban management, and also an important link between ecological environment protection and public health defense. Medical wastewater, whose source and composition are very complex, contains a large number of pathogenic microorganisms, including bacteria, viruses, insect eggs, etc. With the large-scale outbreak and mutation of COVID-19, the safe treatment of medical wastewater is critical to social security and human health. In addition, a large number of antibiotic-resistant bacteria have been detected in medical wastewater due to the misuse of antibiotics (Yu et al., 2021), such as carbapenem-resistant Enterobacteriaceae (Cahill et al., 2019). The direct discharge of medical wastewater will not only cause water and soil pollution, but also cause various diseases that threaten human health (Bibby et al., 2021). Therefore, it is necessary to thoroughly disinfect medical wastewater to prevent the spread of pathogenic microorganisms, thereby protecting public health and ecological environment safety.

At present, traditional disinfection methods are mainly used in medical wastewater treatment, including ultraviolet disinfection, chloride disinfection, and hydrogen peroxide disinfection (Zhang et al., 2020). Among them, ultraviolet disinfection is ineffective in removing drug-resistant bacteria, and cannot continuously disinfect (Islam et al., 2017). The chlorination method (hypochlorite, liquid chlorine, and chlorine dioxide) has the disadvantages of causing secondary pollution easily and being difficult to store and transport (Wang et al., 2020). Due to the unstable nature of peroxides, it is also not convenient for practical medical wastewater treatment. Notably, drug-resistant microorganisms and viruses in medical wastewater cannot be completely eliminated by traditional disinfection techniques (Majumder et al., 2020). Therefore, it is of great significance to explore green and efficient disinfection methods for medical wastewater.

This review focuses on the application of electrochemical disinfection technology in the field of medical wastewater treatment, including electrochemical oxidation, electrolytic chlorination, and the effect of the electric field. The advantages of electrochemical disinfection technology as wastewater treatment are highlighted, and its sterilization mechanism is discussed, which provides useful insights for the development of electrochemical disinfection technology-based treatment of medical wastewater. Furthermore, an electrochemical three-stage disinfection system is proposed for medical wastewater treatment. In the end, we also look forward to the challenges and future progress of electrochemical technology in medical wastewater treatment.

## 2. Electrochemical oxidation

The mechanism of electrochemical oxidation disinfection is to oxidize the microorganisms through the active groups generated by the redox reaction on the surface of the electrode (Giannakis et al., 2021; Hand and Cusick, 2021). Usually, reactive groups are generated by the electrolysis of water and dissolved oxygen in water, such as hydroxyl radicals, ozone, negative oxygen ions, etc. Due to the existence of sulfate ions, chloride ions, and phosphates, corresponding active groups are also generated, including sulfate radicals, chlorine radicals, and phosphate radicals (Giannakis et al., 2021). Various components in microbial cells are oxidized by strong oxidative active groups, which destroy the permeability of the cell membranes, resulting in irreversible changes, and the death of microorganisms (Wang et al., 2019). As reported, hydroxyl radicals generated during anodization are one of the main substances responsible for the inactivation of *Escherichia coli* in a chlorine-free environment (Jeong et al., 2006).

The performance of electrochemical oxidation treatment of wastewater is closely related to electrode materials, current density, hydraulic conditions, and dissolved oxygen (Hand and Cusick, 2021; Zhang et al., 2021). The effect of disinfection is significantly improved when noble metal oxides (such as platinum, ruthenium, iridium, etc.) are used as anode materials. This might be due to the catalytic effect of noble metal oxides on active oxygen and active chlorine, which greatly improves the disinfection capability. It has been reported that titanium electrodes coated with metal oxides are currently mature and stable anode materials (Rathinavelu et al., 2022). When Ti/Sb-SnO_2_/PbO_2_ is used as an anode and the applied current density is 30 mA**·**cm^−2^, wastewater disinfection can be achieved within 12 min, and the energy consumption is only 4.978 kw h m^−3^. The sterilization rate increased with the hydraulic holding time increase and remained stable when the current density was constant. In addition, the ratio of electrode area to flow rate is the ratio of anode working area to wastewater volume, and the appropriate ratio is very important for sterilization. In particular, the increase of dissolved oxygen can increase the concentration of reactive oxygen species in wastewater, thereby promoting to kill pathogenic microorganisms (Rathinavelu et al., 2022).

Importantly, electrochemical oxidation technology could achieve complete disinfection of wastewater without adding any chemicals that could alter the physicochemical properties of wastewater. In addition, electrochemical oxidation technology is not a simple process, which is affected by many factors. For example, chloride ions in solution enhance the destruction of bacteria and viruses by promoting the chain reaction of free radicals (Herraiz-Carboné et al., 2020c). Boron-doped diamond electrodes produce reactive oxygen species at a low voltage of 4–10 V, which can completely kill Enterobacteriaceae and pathogens in wastewater, and significantly reduce spores (Schorr et al., 2019). The pilot test results of the boron-doped diamond electrode also showed a good disinfection effect. In general, the traditional disinfection process based on chlorination treatment is expected to be replaced by electrochemical oxidation technology.

**Figure 1:**
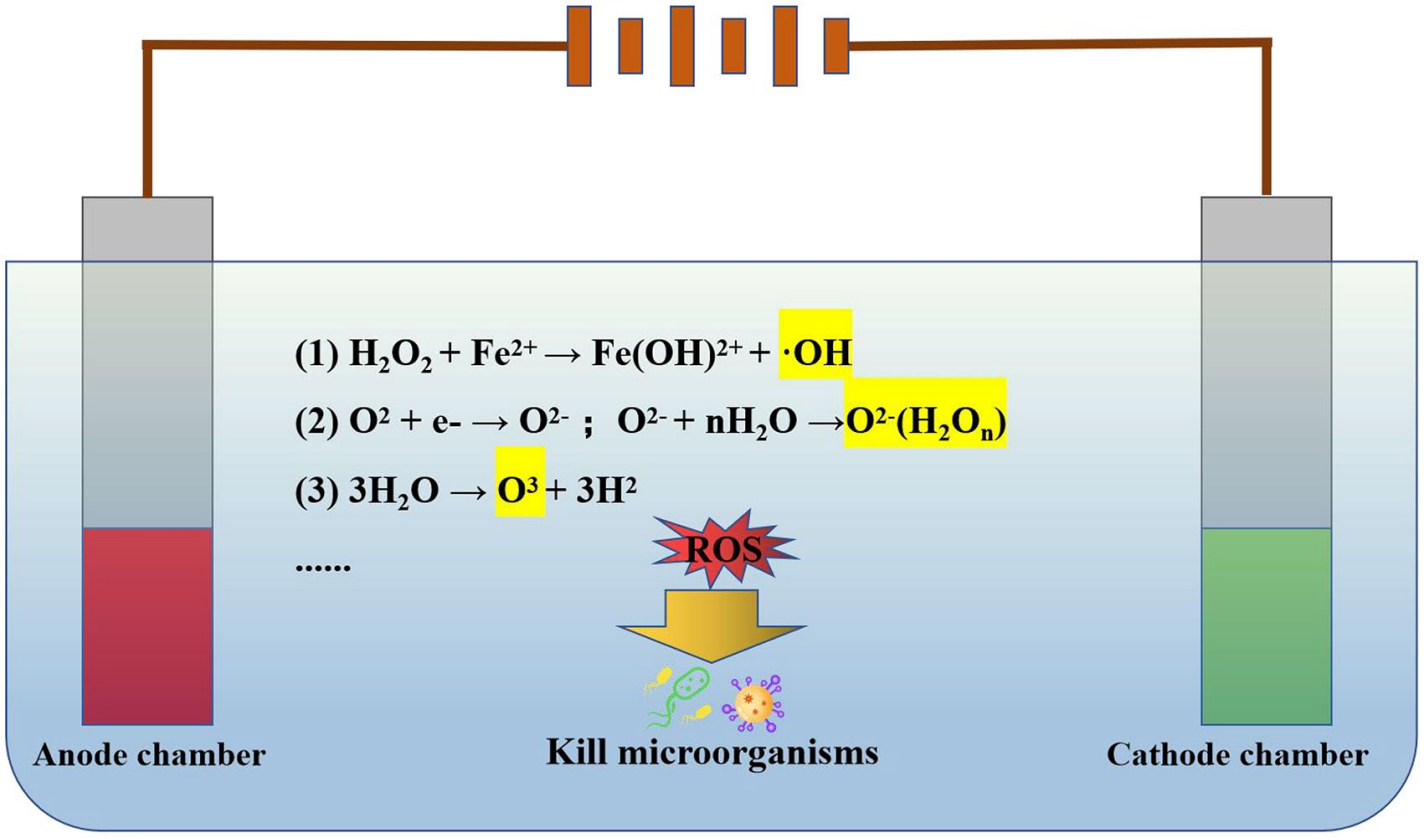
Electrochemical oxidation disinfection mechanism. When electrolyzing water, the redox reaction on the electrode surface can produce hydroxyl radicals, ozone, negative oxygen ions, etc., which can inactivate microorganisms in water by destroying their cell membranes.

In addition, different anode materials lead to differences in sterilization mechanisms during electrochemical oxidation. The boron diamond (BDD) anode promotes the generation of hydroxyl radicals, thereby improving the efficiency of sterilization (Srivastava et al., 2021). Dimensional Stable Anodes enhance the bactericidal effect by increasing the free chlorine generation rate (Feng et al., 2018). *Rhizopus*, *Pseudomonas*, and *Agrobacteria* were effectively killed using mixed metal oxide anodes, which is attributed to the rapid formation of chlorine/hypochlorite (Särkkä et al., 2008). The research shows that the porous SnO_2_ sb reaction membrane electrode (RME) performs very well in the disinfection of wastewater, generates a large number of hydroxyl radicals under the applied voltage of 3.5 V, and realizes 100% removal of *E. coli* and phage MS2, which has practical application potential (Yang et al., 2022). However, in practical application, the concentration of suspended solids in wastewater is high, which is easy to adhere to the surface of RME, thus reducing the disinfection efficiency. Therefore, it is very necessary to add a preprocessing system to remove suspended solids. In particular, the combination of electrochemical oxidation and other methods also shows a good disinfection effect, such as sequential electrocoagulation and electrooxidation treatment system (Heffron et al., 2019), UV-assisted electrochemical oxidation (Wang et al., 2021), photo-assisted electrochemical advanced oxidation process (Herraiz-Carboné et al., 2021). The mechanism diagram of electrochemical oxidation sterilization is as follows (Figure 1).

## 3. Electrolytic chlorination

**Figure 2:**
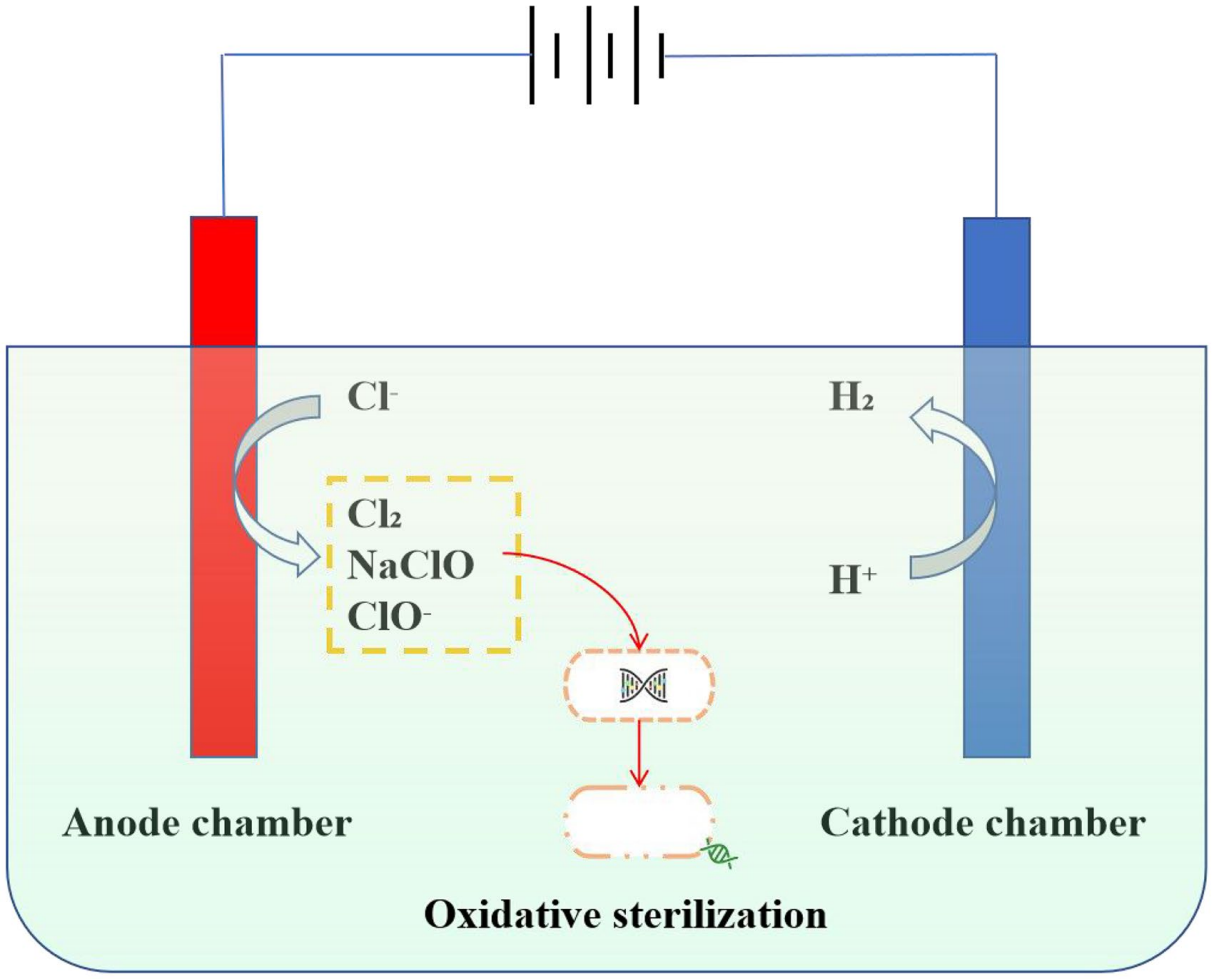
Mechanism diagram of electrolytic chlorination disinfection. Hypochlorite (HClO), which can be rapidly converted from chloride ions by electrolytic chlorination techniques, can pass through cell walls into the interior of bacteria and cause microbial death through peroxidation reactions.

Strongly oxidizing hypochlorite (HClO) could be rapidly converted from chloride ions by electrolytic chlorination technology, which is used for disinfection in wastewater treatment processes (Huang et al., 2016; Carter-Timofte et al., 2021). Due to its small molecular weight, neutrality, and strong oxidizing properties, HClO could pass through the cell wall and enter the interior of the bacteria to directly act on the thiol group of bacterial enzymes. Thus, bacterial death is caused by the destruction of the bacterial enzyme system (Neumann and Rosenheck, 1972; Pang et al., 2021). In addition, HClO destroys the virus shell, nucleic acids, proteins, and enzymes in the virus through an oxidation reaction, resulting in the death of the virus (Bastin et al., 2020; Giarratana et al., 2021). In particular, antibiotic-resistant bacteria (ARB) could also be oxidatively decomposed by electrolytic chlorination. Cotillas et al. (2018) study found that diamond anode electric disinfection can effectively remove antibiotic-resistant bacteria in synthetic urine. Organic substances in urine such as urea can react with active chlorine generated by electrolysis to increase the concentration of chloramine, to prevent the formation of harmful by-products chlorate. Hypochlorite and chloramine produced by electrolysis are the main substances for sterilization, which can be used as the pretreatment of wastewater, It can remove antibiotic-resistant bacteria without producing harmful by-products (Herraiz-Carboné et al., 2020b). In addition, the disinfection efficiency could be effectively improved by the combination of electrolysis and ultraviolet disinfection process. As reported, ARB can be completely removed in all current densities and tested anode materials, electrochemical oxidation with BDD and Mixed Metal Oxides anodes at 50 A**·**m^−2^ can completely remove ARB from urine (Herraiz-Carboné et al., 2020a). Compared with commercial sodium hypochlorite, liquid chlorine, and chlorine dioxide disinfection, electrolytic chlorination has the advantages of safe raw materials, a high degree of automation, and low operating costs (Yan et al., 2021). At present, electrolytic chlorination technology is widely used in wastewater treatment, but there are also some problems, such as high one-time investment and high power consumption (Figure 2).

## 4. Self-action of electric field

The death of microorganisms by electric field self-action (Pulsed Electric Fields, PEF) relies on the breakdown of the cell membrane or electroporation caused by the direct action of the electric field (Elserougi et al., 2016). The thickness of the cell membrane is mechanically compressed under the action of the electric field, disintegrating the cell membrane and the release of the cytoplasm. In addition, cells swell and rupture after the free entry of small molecules into cells, which might be caused by the destabilization of phospholipid bilayers and proteins due to the effect of electric fields (Islam et al., 2017). Under the applied electric field, the radius of the water pores in the cell membrane increased with the pulse duration (Neumann and Rosenheck, 1972). If the radius of induced pores reaches a critical value, the process is irreversible (Weaver and Chizmadzhev, 1996). The cell gradually loses its internal components through this irreversible pore, resulting in cell death (Schoenbach et al., 2000; Heinz et al., 2001). Furthermore, nanowire-assisted electroporation disinfection is an alternative to traditional sterilization methods. Electroporation disinfection cell using three-dimensional copper foam electrodes modified by copper oxide nanowires exhibits the advantages of low operating voltage (1 V), short contact time (7 s), and no chlorine production (Huo et al., 2016). Using triboelectric nanogenerators to drive the copper oxide nanowire electrode array, through the point effect to amplify the electric field, can cause irreversible electroporation to microorganisms. When it is applied to urine polluted by *E. coli*, *Staphylococcus aureus*, *Klebsiella pneumonia*, and *Pseudomonas aeruginosa*, it has high sterilization efficiency (more than 99.9999%), no living chlorine, and simultaneous degradation of organic pollutants, which has great application potential (Zhang et al., 2022).

**Figure 3:**
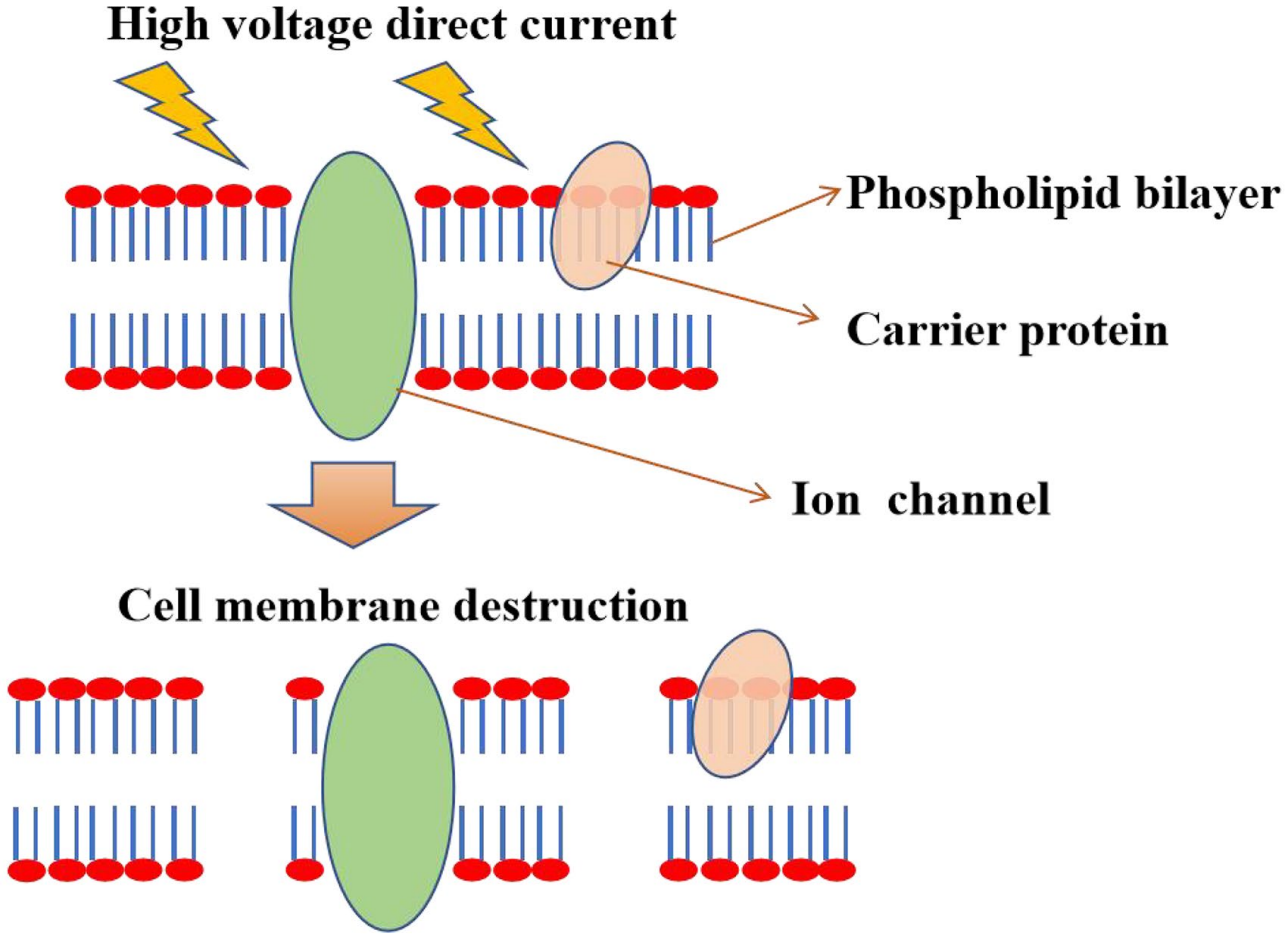
Electroporation mechanism. The electric field action causes destabilization of phospholipid bilayers and proteins, causing cell membrane perforation or rupture and cytoplasmic release, which results in microbial death.

Usually, most hospital wastewater exhibits great genotoxicity before treatment. PEF treatment shows good disinfection effects without side effects and genotoxicity, indicating that PEF is a sustainable method of sterilization (Gusbeth et al., 2009). In particular, PEF could be used as an independent treatment process at critical control points in wastewater treatment. For example, on-site treatment of wastewater generated from hospitals or other healthcare facilities, or in tandem with other conventional methods to reduce the total bacterial load and clinically relevant ARB concentrations. PEF technology could not only kill *Fusarium oxysporum*, but also kill spores of *Bacillus subtilis* in wastewater (Siemer et al., 2014; Zhong et al., 2019). In particular, PEF is also very effective in inactivating suspended microorganisms in wine, beer, and yellow rice wine (Yang et al., 2016). Currently, high energy consumption is a major obstacle for PEF technology, which can be solved by reducing electrode spacing and applying locally enhanced electric fields (Zhou et al., 2021). The mechanism diagram of sterilization by the electric field itself is shown in Figure 3.

## 5. Expectation

In summary, as a new disinfection technology, electrochemical disinfection plays an important role in medical wastewater treatment and on-site epidemic prevention and control. Electrochemical oxidation sterilization has the advantage of high efficiency, but its electrode material is expensive, and a pretreatment system needs to be added to prevent the suspended solids in wastewater from adhering to the electrode surface, resulting in the decline of sterilization efficiency. Electrolytic chlorination has a good effect on removing antibiotic-resistant bacteria, but it also has the problems of a high one-time investment and high energy consumption. The self-action sterilization efficiency of the electric field is high, which can kill bacterial spores and degrade organic pollutants. It can be used as an independent process for on-site medical wastewater treatment (such as shelter hospitals), but it has the problem of high power consumption. Compared with electrolytic chlorination and the electric field itself, electrochemical oxidation technology shows obvious advantages in the sterilization process of medical wastewater, such as low energy consumption, simple structure, and convenient combination with other treatment methods. In addition, the actual medical wastewater is very complex, and the damage and impact of medical wastewater on electrode materials and equipment should also be paid attention to.

To better realize electrochemical disinfection of medical wastewater, it is necessary to improve electrode materials, reactors and reduce power consumption, and integrate existing technologies and processes. For example, the three-stage treatment system of “electric field self-action + electrochemical oxidation + electrolytic chlorination” should be explored. The electric field self-action kills most pathogenic microorganisms through electroporation, and the ones which are not killed will be killed by electrochemical oxidation. Finally, a few pathogenic microorganisms that are not treated by electrochemical oxidation are removed by electrolytic chlorine. The traditional treatment method is expected to be replaced by the above three electrochemical technologies in the disinfection of medical wastewater, to improve the health management level of the city. Overall, electrochemical technology has great potential in the disinfection and treatment of medical wastewater.

## Author contributions

JD, JT, HZ, and YW conceived and wrote the first draft of the manuscript. JD, JT, HZ, YW, JC, and JL revised each part of the manuscript in detail. All authors contributed to the article and approved the submitted version.

## Funding

This study was supported by the Hubei Provincial Administration of Traditional Chinese Medicine Research Project of Traditional Chinese Medicine.

## Conflict of interest

The authors declare that the research was conducted in the absence of any commercial or financial relationships that could be construed as a potential conflict of interest.

## Publisher’s note

All claims expressed in this article are solely those of the authors and do not necessarily represent those of their affiliated organizations, or those of the publisher, the editors and the reviewers. Any product that may be evaluated in this article, or claim that may be made by its manufacturer, is not guaranteed or endorsed by the publisher.
